# Acetyl-CoA the Key Factor for Survival or Death of Cholinergic Neurons in Course of Neurodegenerative Diseases

**DOI:** 10.1007/s11064-013-1060-x

**Published:** 2013-05-16

**Authors:** Andrzej Szutowicz, Hanna Bielarczyk, Agnieszka Jankowska-Kulawy, Tadeusz Pawełczyk, Anna Ronowska

**Affiliations:** 1 Department of Laboratory Medicine, Medical University of Gdańsk, Ul. Dębinki 7, 80-211 Gdańsk, Poland; 2 Department of Molecular Medicine, Medical University of Gdańsk, Gdańsk, Poland

**Keywords:** Acetyl-CoA, Alzheimer’s disease, Cholinergic neurons, Neurotoxins, Pyruvate dehydrogenase, Thiamine deficiency

## Abstract

Glucose-derived pyruvate is a principal source of acetyl-CoA in all brain cells, through pyruvate dehydogenase complex (PDHC) reaction. Cholinergic neurons like neurons of other transmitter systems and glial cells, utilize acetyl-CoA for energy production in mitochondria and diverse synthetic pathways in their extramitochondrial compartments. However, cholinergic neurons require additional amounts of acetyl-CoA for acetylcholine synthesis in their cytoplasmic compartment to maintain their transmitter functions. Characteristic feature of several neurodegenerating diseases including Alzheimer’s disease and thiamine diphosphate deficiency encephalopathy is the decrease of PDHC activity correlating with cholinergic deficits and losses of cognitive functions. Such conditions generate acetyl-CoA deficits that are deeper in cholinergic neurons than in noncholinergic neuronal and glial cells, due to its additional consumption in the transmitter synthesis. Therefore, any neuropathologic conditions are likely to be more harmful for the cholinergic neurons than for noncholinergic ones. For this reason attempts preserving proper supply of acetyl-CoA in the diseased brain, should attenuate high susceptibility of cholinergic neurons to diverse neurodegenerative conditions. This review describes how common neurodegenerative signals could induce deficts in cholinergic neurotransmission through suppression of acetyl-CoA metabolism in the cholinergic neurons.

## General Outline of Brain Energy Metabolism

Brain constitutes for 2 % of human body mass but under resting conditions it utilizes 20 % of whole body oxygen and glucose. Glucose is transported across the blood brain barrier through insulin independent, low-affinity high- capacity, glucose down regulated Glut 1 transporter. Due to the high rate of glucose utilization by brain cells its concentration in CSF is equal to two/third of its level in general circulation. Glucose is an almost exclusive energy substrate in the brain that through glycolytic pathway yields pyruvate, a key precursor of acetyl-CoA, which feeds TCA cycle. The latter is synthesized almost exclusively by pyruvate dehydrogenase complex (pyruvate: lipoate oxidoreductase acceptor acetylating, PDHC) that activities in whole brain, as well as in isolated brain mitochondria are 4–10 times higher than in respective fractions of non excitable tissues. The highest activity of PDHC was found in the hippocampus and the lowest one (60 % lower) in the medulla oblongata [1, 2]. These PDHC patterns did not correlate with differential regional distribution of ChAT activities [2, 3]. It might result form the fact that cholinergic neurons form small about 1 % fractions of entire brain cell population. Therefore, particular interactions between energy and cholinergic metabolism ought to be investigated using models containing greater density of cholinergic neurons.

**Fig. 1:**
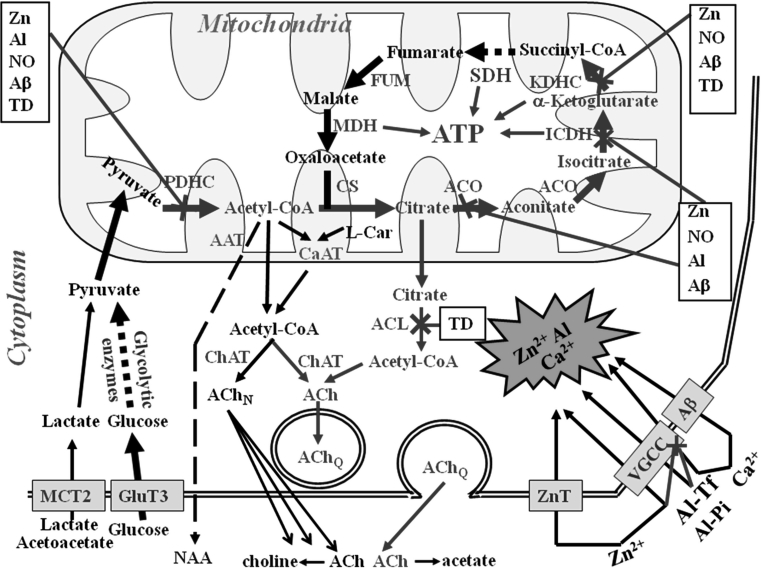
Pathways of acetyl-CoA and energy metabolism in cholinergic neurons of neurodegenerating brain. Combination of diverse neurotoxic signals contributes to Alzheimer’s and other types of cholinergic encephalopathies. They inhibit PDHC and acetyl-CoA synthesis yielding energy deficits of entire population of brain cells. However, cholinergic neurons are more susceptible than others to these neurodegenerative inputs. Cytotoxic-signal induced excessive depolarization of glutaminergic terminals causes increase of [Zn] and [Glu] in the synaptic cleft. Zn, Ca, and chronically accumulated Al enter depolarized postsynaptic cholinergic neurons through ZnTs, VGCC, NMDA and/or Aβ high permeability channels. The increase of cytoplasmic [Ca^2+^] activates nNOS, whereas subsequent everload of mitochondria with this cation inhibits PDHC. Also Zn or NO-derived peroxynitrite radicals acutely inhibit PDHC and other other enzymes of TCA cycle yielding depression of acetyl-CoA synthesis and its utilization for ATP production in the mitochondria. Chronic accumulation of Aβ in extra and intracellular compartments, alone or in combination with Ca and other metals alters multiple transport entities in the plasma and mitochondrial membranes and directly inhibits PDHC and aconitase. The prolonged depolarization and energy deficts increase nonquantal ACh release and inhibit its quantal Ca-dependent release. These conditions limit direct or ACL-dependent transport acetyl-CoA from mitochondria to the site of ACh synthesis in the synaptoplasmic compartment. The decrease of acetyl-CoA in the synaptoplasm causes instant inhibition of ChAT, yielding depression of ACh pool and rates of its quantal release. Prolonged decrease of cytoplamic [acetyl-CoA] results in adaptative suppression of ChAT expression aggravating deficits of cholinergic neurotransmission. Part of acetyl-CoA pool is utilized for intramitochondrial NAA synthesis, which is subsequently released out of the neuron. Decrease of mitochondrial acetyl-CoA results in energy deficits, and correlates with death rate of cholinergic neurons. *Red arrows* activation, *blue arrows* inhibition, *green letters* enzymes, *black boxes* neurotoxic agents (Color figure online)

Neurons that constitute about 10 %, of brain cells consume 70 % of glucose and oxygen supplied to this organ. That is due to the fact that neurons, possess high density/high glucose affinity Glut 3 transporters and their in situ, rates of metabolic fluxes of high-energy intermediates that are at least 20 times faster than in glial cells (Fig. 1) [4]. Brain may also utilize acetoaetate/β-hydroxybytyrate as a complementary source of acetyl-CoA [5, 6]. In physiological conditions levels of these ketoacids in extracellular fluids are well below 0.05 mmol/L. However, under pathologic conditions, such as diabetes, their extracellular concentrations may reach levels as high as 5 mmol/L. At such concentrations, ketoacids enter the brain through MCTs competing effectively with lactate (Fig. 1). Under in vitro conditions, these ketoacids were capable maintaining normal levels of ATP and phosphocreatine in different brain preparations. Moreover, in diabetic-ketotic rat brain ketoacids along with glucose or pyruvate caused marked rises acetyl-CoA, ACh synthesis and release from isolated nerve terminals [7].

Under in vitro conditions, neuronal cells utilize lactate/pyruvate as preferred energy substrates. The increase of lactate in circulation during exercise or pathologic hypoxia may cause several fold increase of its transport through the BBB. Inside the cells lactate remains in equilibrium with pyruvate due to very high activity of lactic dehydrogenase. Therefore, the net rate of its flux to pyruvate depends on utilization rate of the latter in PDHC and pyruvate carboxylase reactions. Pyruvate through PDHC reaction is almost exclusive source of acetyl-CoA in neurons and other types of brain cells (Fig. 1). However, anaplerotic reaction of pyruvate carboxylation to oxaloacetate by pyruvate carboxylase occurs mainly in astrocytes [8]. Therefore, oxaloacetate has to be transported into the neurons through indirect oxaloacetate-aspartate and glutamine-glutamate shunts. Both metabolites are required by citrate synthase to feed TCA cycle.

Also astrocytes produce large amounts of lactate. Fraction of this metabolite is taken up and utilized by the neurons thanks to high expression of MCT in the neuronal plasma membranes, and PDHC activity in their mitochondria [9]. Therefore, the astrocyte-derived lactate is postulated to be an important precursor of acetyl-CoA and oxaloacetate-substrates for condensation reaction and energy production in neurons. Results of in vitro experiments on brain slices, synaptosomes and cultured neuronal cells demonstrate that lactate/pyruvate with l-malate are better energy substrates and precursors of acetyl moiety for ACh synthesis than glucose [10].

On the contrary, under in vivo conditions blood lactate, even in high concentrations, could not fully replace the glucose, despite of robust expression of MCT on the blood brain barrier. Only during the suckling period brain is capable to extract lactate from circulation [11]. The source of discrepancies between in vivo and in vitro experiments remains unknown.

Neurons have no capacity for acetate utilization. However, they synthesize large amounts of *N*-acetyl-*N*-aspartate (NAA) in aspartate-CoA transferase (acetyl-CoA:l-aspartate *N*-acetyltransferase, EC 2.3.1.17) reaction located in their mitochondria. Neurons contain 98.7 % fraction of overall brain pool of this metabolite (Fig. 1). Its intraneuronal concentration reaches levels 20 mmol/L [12]. NAA is transported to oligodendrocytes, where it may be hydrolysed to acetate by aspartoylacylase (aminohydrolase II, EC 3.5.1.15) and converted back to acetyl-CoA by acetyl-CoA synthetase (acetate:CoA ligase AMP, EC 6.2.1.1).

**Fig. 2:**
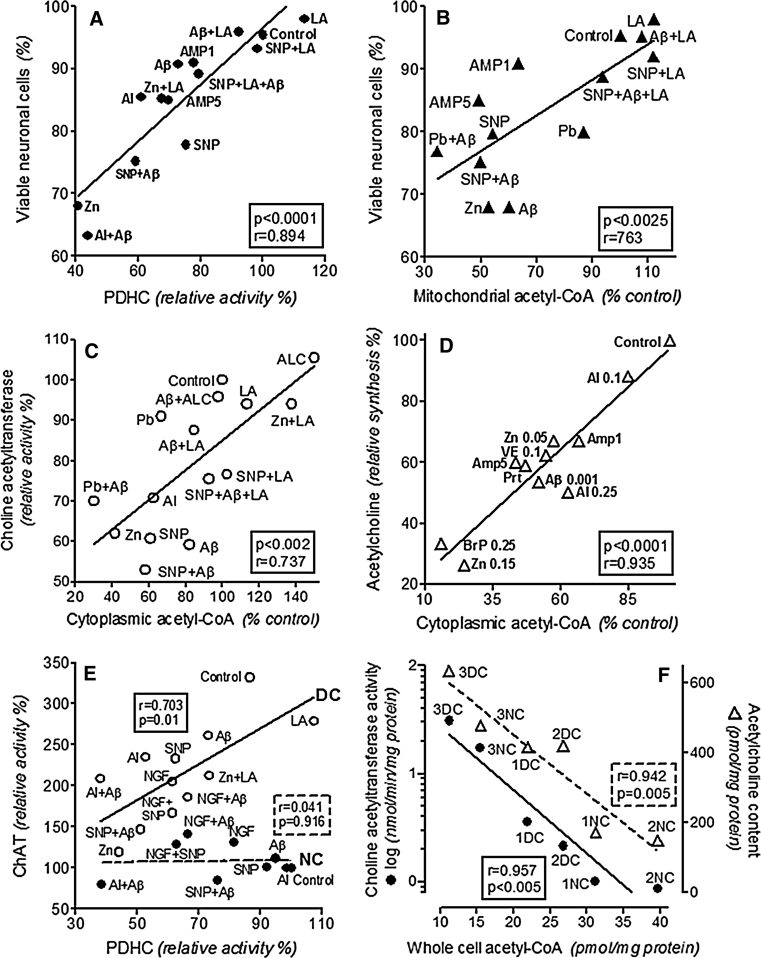
Selected correlations between different parameters of energy and ACh metabolism in differentiated SN56 cholinergic neuronal cells and brain nerve terminals under various neurotoxic and neuroprotective conditions (**a**–**d**): **a** significant correlation between cell viability and PDHC activity; **b** significant correlation between cell viability and acetyl-CoA level in their mitochondria; **c** significant correlation between cytoplasmic acetyl-CoA level and ChAT activity; **d** significant correlation between ACh synthesis and cytoplasmic acetyl-CoA level; **e** significant correlation between PDHC and ChAT activities in differentiated cholinergic cells and lack of such correlation in nondifferentiated ones; **f** significant inverse correlations between ChAT activities (*circles*)/ACh contents (*triangles*) and whole cell acetyl-CoA levels in different genetically and postranslationally modified phenotypes of cholinergic SN56 cells. (1) Native differentiated (DC) and nondifferentiated (NC) SN56 cells; (2) TrkA expressing T17 NC/DC SN56 cells; 3. 2ChAT overexpressing NC/DC SN56 cells. Data recalculated from: [27, 35–38, 45, 54–56, 79, 131]

Overall NNA concentration in the brain is in the range of 10 mmol/kg. Therefore, its signal in the brain can be easily detected by PET and used as marker of viability of brain neurons. The levels of NAA were found to be decreased in the specific regions of cerebral cortex in various brain pathologies including: Alzheimer’s, Parkinson’s diseases as well as Wernicke, dialysis and aluminum encephalopathies [12]. In each of these pathologies the PDHC activity and parameters of energy metabolism were found to be decreased. Experiments on animal and cellular models of these encephalopathies demonstrated inhibition of PDHC that correlated with suppression of acetyl-CoA levels in the mitochondrial compartment and the rate of cell death (Figs. 1, 2) [1, 6, 13–15]. It might directly decrease the rate of NAA synthesis in the aspartate acetyltransferase reaction. However, no direct correlation studies are available.

Oral administration of large doses of glyceride triacetate was reported to cause marked increases of acetyl-CoA and phosphocreatine levels in the rat brain [16]. This rise was linked with attenuation LPS-induced inflammatory reactions in microglia and improvement of energy metabolism in glial cells [16, 17].

Total number of astrocytic, microglial and oligodendroglial cells in the brain is 10 times greater than that of the neurons, but their oxidative metabolism utilizes only 30 % of the brain glucose. They display about 4 times lower activities of PDHC, ketoglutarate dehydrogenase (2-oxoglutarate:lipoate oxidoreductase acceptor acylating, EC 1.2.4.2., KDHC) and other enzymes of oxidative metabolism than neurons [18, 19]. High level of phosphorylation of E1 subunit of PDHC in astrocytes, could also inhibit pyruvate consumption in this compartment [20]. On the contrary, in astrocytes rates of glucose and lactate uptake are 2–4 times faster than in isolated neurons. Therefore, astrocytes may release both lactate and glucose to support neuronal energy metabolism [21, 22]. Accordingly, oxidative metabolism in neurons, but not in astroglia, was strongly activated by pyruvate and lactate. It gives rise to the hypothesis that under hypoxic/hypoglycemic conditions lactate may replace glucose as a principal energy substrate [21, 23].

Astrocytes also avidly take up glutamate, released from glutamatergic terminals, through EAAT1-2 transporters and convert it to l-glutamine in Pi-activated glutamine synthetase reaction [24]. Thereby, they protect postsynaptic neurons against excitotoxic injury, by excess of extracellular l-glutamate. In addition, astrocytes contain high activities of pyruvate carboxylase yielding production large amounts of oxaloacetate [8]. They supply these metabolites to the neurons in form of glutamine and aspartate, through glutamine-glutamate and aspartate shuttles, respectively [8]. Most of compartmentalization studies were performed on isolated primary neuronal or glial cell cultures, to deal with possibly homogenous cellular populations [24]. One should, however keep in mind that these cultures are still heterogeneous. For instance, primary neuronal cultures consist of neurons of different transmitter systems with dominating fraction of glutamatergic ones.

However, activities of PDHC in different brain regions displayed no correlations with distribution specific markers of cholinergic pericarya and/or terminals [2, 3, 25]. Also, differentiation of SN56 cholinergic cells was accompanied by the decrease but not increase of PDHC activity [26, 27]. It indicates that rate of acetyl-CoA synthesis by PDHC in cholinergic neurons is not positively linked with expression their transmitter phenotype (Fig. 1). On the other hand, during postnatal development the increase of ChAT activity in cerebrum was accompanied by respective elevations in PDHC levels, what might be compatible with maturation-induced activation all transmitter systems [3, 28, 29].

### Age Dependent Modifications

Brain maturation stimulates glucose uptake, glycolysis and oxidative pathways due to increased expression of respective genes and enzyme levels. These alterations display temporal correlations with maturation-dependent elevations in brain cholinergic and glutamatergic transmitter metabolism, both requiring an adequate provision of acetyl-CoA by PDHC and its flux through TCA [28, 30, 31]. On the other hand, physiologic brain aging is characterized by continuous, although relatively small, decline of structural and metabolic markers of cholinergic neurons and their key physiological neurotransmitter functions. Also, no alterations in activities of key enzymes of oxidative metabolism including PDHC, KDHC, aconitase and other enzymes of TCA cycle and respiratory chain were found [25]. However, in these conditions deficits in several antioxidative species such as glutathione, carnitine and lipoate along with excessive glutamatergic signaling, and free radical production appeared [32–34]. That, made aged brains increasingly vulnerable to common pathologic conditions such as hypoxia, hypoglycemia, excitotoxic stimulation, amyloid-β accumulation, Zn, Fe, Al excess and thiamine (TD) or other vitamin deficiences (Fig. 1). All these pathologic signals strongly inhibited PDHC, KDHC, aconitase [citrate (isocitrate)hydrolase, EC 4.2.1.3.] and other key enzymes of acetyl-CoA and energy metabolism (Fig. 1) [14, 27, 35–38]. It might cause fast decay of cholinergic neurons located in the septal nuclei (Fig. 2a, b), thereby aggravating age-dependent physiologic limitations of memory and cognitive functions [39]. In addition, other studies on 24 months rats revealed marked 40 % decrease of PDHC activity, with concomitant increases in expression and activity of pyruvate dehydrogenase kinase (ATP:[pyruvate dehydrogenase (acetyl-transferring)] phosphotransferase, EC 2.7.11.2.), yielding hiperphosphorylation of PDHC-E1 subunit. That, could be responsible for decreased levels of ATP and increased accumulation of lactate in senescent brains [40].

### Key Role of Acetyl-CoA

Acetyl-CoA is a key energy precursor-intermediate in all cells of our organism. In the brain it is almost exclusively synthesized in the mitochondria in PDHC reaction providing 97 % of energy (Fig. 1). Brain does not utilize fatty acids as a source of acetyl-CoA. However, during systemic ketosis it may utilize β-hydroxybutyrate-derived acetoacetate through acetoacetyl-CoA synthestase, succinyl-CoA acyltransferase, and acetoacetyl-CoA thiolase reactions. Irrespective of the origin, acetyl-CoA is efficiently incorporated into TCA cycle through high activity citrate synthase step. Its equilibrium constants equal to 2.2 × 10^8^ shifts this reaction far toward citrate formation [41]. It causes the level of acetyl-CoA in brain mitochondria being low and strongly dependent on the rate of its synthesis by PDHC (Fig. 1). Therefore, any conditions suppressing PDHC activity decrease acetyl-CoA level in the brain mitochondria. Significant correlations between PDHC activity and acetyl-CoA levels have been reported in our studies on different models of neurodegeneration (Fig. 2a, b) [6, 12, 42–44].

In last 40 years number of studies directly assessed acetyl-CoA content in different brain preparations [7, 43, 45–55]. However, majority of them did not take into account apparent differences in acetyl-CoA distribution between mitochondrial and cytoplasmic compartments in cholinergic and noncholinergic neurons as well as in glial cells. Only experiments performed either on brain nerve terminals or in clonal cholinergic neuronal SN56 cell lines assessed subcellular distribution of acetyl-CoA, so far [14, 36, 38, 45, 56]. These models enabled one to investigate acetyl-CoA shifts between mitochondrial and cytoplasmic compartment in pure cholinergic cells under different physiologic and pathogenic conditions (Figs. 1, 2).

Small fraction of mitochondrial acetyl-CoA, not used in TCA cycle, serves as a source of acetyl units for different synthetic processes taking place in cytoplasmic compartment of these neuronal cells (Fig. 1). However, in resting neurons inner mitochondrial membrane is relatively impermeable for acetyl-CoA and other acyl-CoA esters [6, 57]. Therefore, acetyl units are transported to cytoplasm indirectly, in the form of citrate, acetyl-l-carnitine, or acetate (Fig. 1) [57]. Citrate leaves mitochondria through dicarboxylate transporter to be cleaved by ATP-citrate lyase (acetyl-CoA:oxaloacetate *C*-acetyltransferase [(*pro*-*S*)carboxymethyl-forming, ADP phosphorylating, EC 2.3.3.8, ACL) to acetyl-CoA and oxaloacetate. Studies with specific inhibitor of ACL (−) hydroxycitrate revealed that this pathway provides about 30–50 % of acetyl units in the cytoplasmic compartment of both neuronal and glial cells [44, 45, 58]. On the other hand, carnitine acetyl transferase system (acetyl-CoA:carnitine *O*-acetyltransferase, EC 2.3.1.7) in the brain mitochondria was found to be active only in the presence of excess of exogenous acetyl-l-carnitine (ALC) [59, 60]. Also immature brains were reported to take up and metabolize ALC from the blood more avidly than the adult ones [61].

Studies on whole brain mitochondria of mixed glial-neuronal origin have demonstrated that pathophysiologically relevant [Ca^2+^] of 10^−6^ mol/L concentrations, evoked direct release of acetyl-CoA (Fig. 1) [14, 45, 50]. Also in synaptosomes adequate fraction of their mitochondrial acetyl-CoA was found to reach cytoplasm directly through Ca-activated, verapamil-inhibited pathway (Fig. 1) [45, 56]. In primary neurons depolarization increased cytoplasmic [Ca^2+^], and opened reversible, high permeability transition anionic channels (PTP) through which acetyl-CoA moieties might diffuse from mitochondria to cytoplasm, down the concentration gradient [36, 62].

In addition, acetyl-CoA may leave mitochondria as NAA. [12]. Concentrations of substrates for aspartate *N*-acetyltransferase, aspartate and acetyl-CoA, are estimated to be about 1.0 and 0.12 mmol/L [36], whereas corresponding Km values are equal to 0.58 and 0.058 mmol/L, respectively [36, 63]. Therefore, pathologies that depress acetyl-CoA formation by PDHC in the brain are likely to decrease its NAA levels. Indeed, the loss of NAA was observed in brain PET scans of AD and PD patients [13, 64]. Neurons cannot utilize NAA as a source of acetate as they contain no aspartoylhydrolase in their cytoplasm. Therefore, NAA may serve as an acetyl moiety donor after its release to extracellular space followed by its uptake by oligodendroglia. The metabolic flux of acetyl units through this pathway was estimated to be equal to 2.5–3.0 % of rates of glucose to pyruvate and 1.2 % pyruvate to acetyl-CoA conversion rates, respectively [12, 36].

These data indicate that there is an intra and intercellular compartmentalization of acetyl-CoA in the brain. Acetyl-CoA in each cellular compartment of the brain forms two metabolically linked but functionally independent pools (Fig. 1). Brain mitochondria are the primary compartment for acetyl-CoA synthesis by the PDHC. Over 90 % of acetyl-CoA is subsequently utilized for energy production. Thus, availability of this metabolite in mitochondria may be a key factor determining viability of neuronal and other cell types in brain (Figs. 1, 2b) [27]. Concentration of acetyl-CoA in the mitochondrial matrix is 5–7 times higher than in cytoplasm and might determine rates of its transport to latter compartment [36]. On the other hand, the influence of cytoplasmic acetyl-CoA itself on velocity of different synthetic pathways seems to be independent of the mitochondrial pool (Fig. 2e) [38, 65].

## Age-Dependent Alterations of Brain Acetylcholine Metabolism

### Cholinergic Transmission

The cholinergic transmission is performed by specific groups of neurons, which synthesize ACh in cytoplasmic compartment, accumulate in synaptic vesicles and release it into the synaptic cleft in a quantal, Ca-dependent mode. Neurotransmitter binding with subsequent activation of postsynaptic muscarinic and nicotinic receptors is connected with cognitive functions, emotional reactions as well as short and long term memory formation in humans and animals.

ACh is synthesized by choline acetyltransferase (acetyl-CoA:choline *O*-acetyltransferase, EC 2.3.1.6., ChAT) from acetyl-CoA and choline in cytoplasmic compartment of cholinergic neuron axonal terminals which equilibrium constant equal to 13 favors formation of this transmitter:

1 $${\text{choline}} + {\text{acetyl-CoA}} \rightleftarrows {\text{ACh}} + {\text{CoA}} $$

The release of ACh creates out of equilibrium conditions in transmitter synthesizing compartment and triggers ChAT reaction. There is thought, that transmitter functions are executed by ACh synthesized in cholinergic nerve terminals and accumulated in synaptic vesicles and released in a quantal mode in response to their depolarization. The content of ACh in cholinergic neurons is maintained on quite stable level despite of marked variations in their activity [6, 66]. It indicates that ChAT activity in cholinergic neurons in situ is high enough to prevent ACh depletion during their activity. Levels of acetyl-CoA and choline in cytoplasm of cholinergic neurons were found to be much below their optimal concentrations used in assay media [6, 66]. Therefore, the rate of ACh synthesis under out of equilibrium conditions, induced by the evoked transmitter release, may be regulated by both ChAT protein level, as well as by substrates availability.

Both ChAT and VAChT are recognized specific structural markers for cholinergic neurons. Their levels depend on the expression of *CHAT* and *VACHT* genes sharing common cholinergic locus regulated by promoters, activated by CREB and retinoic acid receptors (RAR) [67]. Also nerve growth factor (NGF) was found to induce differentiation of septal cholinergic neurons through multiple signaling pathways linked with TrkA, high affinity surface receptors and suppressed through low specificity p75 receptors [27, 54, 55, 68]. Activation of these pathways leads to increased expression of ChAT, VAChT, high affinity choline transport system (CHT1) yielding morphologic maturation and activation of ACh metabolism [54, 69, 70].

Both in ageing animals and in humans decrease in cortical ACh metabolism precedes those in ChAT and VAChT expression [6, 71–73]. In rats, loses in these cholinergic markers, as well as decreased density of cholinergic neurons in the septum, and progressive cognitive deficits appeared several months later. However, in senescence-accelerated, 8th and 12th month old mice, loses in activity and mRNA for ChAT in hippocampus and cerebral cortex as well as decreases in density of septal cholinergic neurons significantly correlated with deterioration their spatial cognitive tests [74]. There are suggestions that age-linked neurodegeneration may be caused by disequilibrium between TrkA and p75NTR receptors [75].

### Metabolism of Acetyl Moiety of Acetylcholine

There is little data on regulation of acetyl-CoA metabolism in brain cholinergic neurons. It results from the fact that cholinergic neurons constitute about 1 and 10 % of whole brain cell and nerve terminal populations, respectively [76]. Therefore, any conclusion concerning pathologic alterations in acetyl-CoA metabolism in cholinergic neurons, are drawn with assumption, that changes of this metabolite in cholinergic and noncholinergic compartments remain the same. However, that may not be the case. For instance, clonal SN56 cholinergic cells from mice septum, with low and high expression of the cholinergic phenotype, had higher and lower levels of acetyl-CoA in their mitochondria, respectively (Fig. 2f) (Table 1) [38, 54, 77].

**Table 1:** Studies describing preferential energy/acetyl-CoA dependent susceptibility of cholinergic neurons to neurodegenerative signals

Experimental model	Neurotoxin/mode of application/level	Parameter—relative effect of neurotoxin						References
11–13 months old	Aβ_1–42_ brain level (nmol/kg)	(% of wild type sibilings)						[171]
		Glucose utilization				Learning		
FVB/N mice-brain	0.175	CtxT 67, CtxO 64, Inf.col. 98				25		
WT sibiling mice-brain	<0.002	100				100		
RN46 cell line culture	Aβ_25–35_ added 48 h (mmol/L)	MTT reduction rate (% control)						[151]
CNTF-cholinergic diff.	0.01	56						
BDNF-serotoninergic	0.01	100						
SN56 cholinergic cells culture	Aβ_25–35_ added 24 h (mmol/L)	Relative effects-% (+) increase (−) decrease						[55]
		TP (+)	acetyl-CoA_mit_	acetyl-CoA_cyt_		ChAT		
Nondifferentiated	0.001	+11	−8	−10		−10		
Differentiated	0.001	+31	−40	−39		−64		
Primary neuronal culture	Aβ_25–35_ added 24 h (mmol/L)	Relative effects-% (+) increase (−) decrease						[152]
		MTT reduction		caspase 3activation		[Ca_i_]		
Hippocampus	0.025	−86		+89		+88		
Cortex	0.025	−42		+33		+38		
17 months old brain synaptosomes	Aβ_1–42_ brain level (mmol/kg)	Relative values (% of WT sibilings)						[165]
		Pyruvate util.		acetyl-CoA_mit_		ChAT	ACh content	
Tg2576 mice	0.008	60		71		106	60	
WT sibilngs	n.d.	100						
Primary neuronal culture	SNAP added 24 h (mmol/L)	Relative effects-% (+) increase (−) decrease						[135]
		TUNEL+					ChAT	
Medial septum	0.5	+69					−60	
Brain stem	0.5	+21					0	
SN56 cholinergic cells culture	SNAP added 24 h (mmol/L)	Relative effects-% (+) increase (−) decrease						[137]
		TUNEL+					LDH release	
Nondifferentiated	1.0	+12					0	
Differentiated	1.0	+34					+31	
SN56 cholinergic cells culture	SNP added 10 min (mmol/L)	Relative effects-% (+) increase (−) decrease						[15, 79]
		TB (+)		PDHC		acetyl-CoA_tot_	ChAT	
Nondifferentiated	1.0	+15		−8		0	+1	
Differentiated	1.0	+27		−28		−42	−30	
	Aβ_25–35_ 24 h + SNP 10 min added							
Nondifferentiated	0.001 + 1.0	+23		−15		n.d.	−24	
Differentiated	0.001 + 1.0	+37		−56		−50	−41	
Cholinergic cell lines culture	SNAP added 24 h (mmol/L)	Relative effects (fold)						[134]
		P-ERK_2_					DNA fragmentation	
Medial septum-derived	0.1	2.1					14	
Brain stem-derived	0.1	1.2					10	
SN56 cholinergic cells culture	Zn added 30 min (mmol/L)	Relative effects-% (+) increase (−) decrease						[38]
		TB (+)	acet-CoA_mit_	acet-CoA_cyt_		ChAT	ACh_ctn_	
Nondifferentiated	0.2	+22	−23	−100		−25	−68	
Differentiated	0.2	+42	−53	−88		−17	−141	
SN56 cholinergic cell culture	Cell clone	Relative values-fold of nondifferentiated wild type						[77]
		ChAT		PDHC		acetyl-CoA_total_	ACh_content_	
Nondifferentiated	Wild type SN56	1.0		1.0		1.0	1.0	
Differentiated	Wild type SN56	3.59		0.86		0.72	2.20	
Nondifferentiated	SN56ChAT2	17.2		0.92		0.38	2.17	
Differentiated	SN56ChAT2	30.9		0.86		0.27	2.89	
SN56 cholinergic cell culture	Amprolium added 48 h. (mmol/L)	Relative effects-% (+) increase (−) decrease						[131]
		TDP	TB (+)	Pyruvate_util_		acetyl-CoA_tot_	ACh_ctn_	
Nondifferentiated	2.0	−31	+5	−27		−37	−18	
Differentiated	2.0	−31	+13	−44		−46	−33	

Aβ, amyloidβ peptides; ACh, acetylcholine content; ChAT, choline acetyltransferase activity; CxT CxO, temporal, occipital cortex; FVB/N mice, MTT, methyl tertrazoliun salt reduction rate; PDHC, pyruvate dehydrogenase complex activity; SNAP, S-Nitroso-*N*-Acetyl-d,l-Penicillamine; SNP, sodium nitroprusside; SN56ChAT2, ChAT gene transfected septal cholinergic hybride neuroblastoma cells SN56.B5.G4; TB(+), trypan blue retaining cells; TDP, thiamine diphosphate; Tg2576 APP695 double mutation K670N, M671L mice; WT, wild type cells or mice

Acetyl-CoA for synthesis of transmitter pool of ACh is synthesized almost exclusively inside of cholinergic neuron mitochondria by PDHC from glucose-derived pyruvate (Fig. 1). In ketotic diabetes part acetyl units for ACh synthesis may be provided by acetoacetate/β-hydroxybutyrate [5, 7]. It is estimated, that only 1–3 % of acetyl-CoA pool synthesized in cholinergic neurons is utilized for ACh production. Despite of that there is a tight interdependence between pyruvate oxidation and ACh content and rate of release both in whole brain, isolated synaptosomes as well as in cultured cells [15, 35, 78, 79]. That may be due to the competition for acetyl-CoA between high velocity TCA and relatively slow rate transporting pathways (Fig. 1) (Table 1).

Among several pathways of acetyl group transfer through the mitochondrial membrane (see “Key Role of Acetyl-CoA”) only two seem to be significant sources of acetyl-CoA for ACh synthesis in the cholinergic neurons. They are: ACL pathway, providing 20–50 % of acetyl units, and the direct efflux of acetyl-CoA through Ca-dependent reversible PTP (Fig. 1) [44, 45, 50].

In addition, ACL pathway provides acetyl-CoA for acetylation of histones and different structural proteins of various mammalian cells including neurons [65]. Thereby, acetyl-CoA-dependent disturbances in protein acetylation may contribute to epigenetic pathomechanisms of Alzheimer’s and other neurodegenerative diseases [80, 81].

During postnatal development of cerebrum activity of ChAT increased several-fold, but the ACL activity remained on stably high level. On the other hand, in cerebellum containing no cholinergic elements, activity of ACL was decreased by about 70 % [3, 31]. Significant correlations have been found between ChAT and ACL activities in different regions of the brain, and in lesioned hippocampus [82]. Also, differentiation of cholinergic S-20 but not NIE-115 dopaminergic neuronal cells caused twofold increase of ACL activity [83].

There is generally accepted that in resting cells in situ, mitochondrial inner membrane is impermeable to acetyl-CoA [45, 57]. However, depolarization-induced increase of cytoplasmic Ca^2+^ may generate reversible PTP, allowing transient direct release of acetyl-CoA from mitochondria to cytoplasm [45, 62, 84]. Studies with Ca chelators and Ca-channel antagonists indicate that up to 60–70 % of acetyl-CoA for ACh synthesis is transported out of mitochondria by the direct release [56].

ALC is thought to be another alternative source of acetyl units for brain ACh synthesis. Under in vivo conditions systemic application of ALC was found to improve energy and ACh metabolism in the brain, as well as cognitive performance of aged or pharmacologically depressed animals [60, 85, 86]. In fact, dietary supplementation of mice with ALC attenuated pentylenetetrazole-induced seizures through restoration normal levels of ATP and NAA [87]. Beneficial effects of ALC supplementation might result from general improvement of energy metabolism and synthesis of structural lipids but not from specific influence on neurotransmission in cholinergic neurons [86, 88]. However, none of these reports assessed acetyl-CoA content in ALC treated brains. In turn, our data demonstrate that addition of ALC to cultured cholinergic neuroblastoma cells increased acetyl-CoA level in their cytoplasm and prevented A-β induced suppression of ACh metabolism (Fig. 2c, d) [55].

Direct interactions between acetyl-CoA and ACh metabolism were demonstrated by studies on nondifferentiad and cAMP/retinoic acid or NGF-differentiated cholinergic SN56 neuroblastoma cells (Fig. 2c, d). They revealed that differentiation-evoked elevations in their ChAT and ACh were accompanied by suppression of acetyl-CoA level in their mitochondria and its increase in the cytoplasmic compartment (Fig. 2f) [54, 55].

## Early and Late Mechanisms of Energy and Acetyl-CoA-Dependent Disturbances in Cholinergic Encephalopathies

Principal clinical symptoms of AD and other cholinergic encephalopathies include progressive cognitive deficits leading to dementias, frequently combining with motor disability [89–91]. They correlate well with the degree of functional and structural loses of basal forebrain cholinergic neurons projecting axons to hippocampus and different cortical areas [92]. Another key feature of these pathologies is the suppression of energy metabolism, which correlates with losses of cholinergic markers in affected areas of brain cortex or spinal cord segments. Dysfunction of brain mitochondria is thought to be both the consequence of pathologic insults as well as a source of signals triggering neurodegeneration [93–95]. There is a hypothesis, that particular susceptibility of cholinergic neurons to degeneration results from the fact that their acetyl-CoA, synthesized in mitochondria by PDHC is used not only for energy production in TCA cycle and respiratory chain but also for ACh synthesis in the cytoplasmic compartment (Fig. 2a, b, f) [6, 27, 78, 96].

### Alzheimer’s Disease

Alzheimer’s disease (AD) is the most frequent form of dementia in senescent population. Estimated number of patients suffering of this pathology reaches 25 million worldwide [97]. AD is a clinical-phenotypic presentation of various sporadic and inherited diseases. Immunohistochemical examinations of autopsied brains reveal accumulation of extracellular amyloid-β (Aβ) deposits and neurofibrillary tau protein type degeneration inside the neurons. The amyloidogenic form is Aβ_1–42_ containing 42 aminoacids, originates from proteolytic processing of transmembrane amyloid precursor protein (APP), by the amyloid-β cleaving enzyme (BACE1, β-secretase). Dominating product of APP proteolysis by ADAM (α-secretases) is 26 aminoacid non-amyloidogenic peptide P3 (Aβ_17–42_ fragment) [98, 99].

The large fraction of sporadic AD patients is carrying double copies of *APOE4* alleles [100, 101]. Moreover, these patients display significant associations with down-regulation of multiple transcripts of genes involved in oxidative phosphorylation, energy metabolism as well as with synaptic vesicles docking and fusion [101–103]. AD patients, being *APOE4* carriers displayed strong correlation between suppression of KDHC, PDHC and ChAT activities in brain cortex and cognitive dysfunction rate [13, 104]. Reduced activities of PDHC and KDHC in autopsied AD brains displayed strong inverse correlations with clinical dementia rating [13, 105]. Also, glucose Glut 1 and 3 transporters density was reduced in affected brain areas, as demonstrated by in vivo PET and in *post mortem* cytochalasin B binding studies [106, 107].

The key biochemical and histopathologic finding in AD brains is preferential loss of cholinergic neurons located in septal nuclei, and their axonal projections to hippocampus and several areas of brain cortex. The decreases of several cholinergic markers such as ChAT, CHT1, VAChT activities and protein levels were observed *post mortem* in affected brain areas [71, 92, 105, 108]. The loss of cholinergic markers correlated with both suppression of energy metabolism and severity of dementia shown shortly before patient’s death [13, 92, 108]. Neuronal loss has been consistently shown in key cholinergic brain nuclei in sixty-seven studies over 20 year period [108]. On the other hand, several noncholinergic transmitter systems were not or much less suppressed by these conditions [109, 110, 111].

Several putative pathomechanisms are involved in AD encephalopathy. They include: Aβ_1–42_ accumulation, excitotoxicity/NO excess, mitochondrial dysfunction, hypoxia/anoxia/hypoglycaemia, oxygen radicals formation, inflammation, metals (Ca^2+^, Zn^2+^, Fe^2+^, Al^3+^) accumulation, or neurothrophin depletion, which were investigated as cytotoxic signals in AD (Fig. 2) [27, 90, 91, 93, 94, 112–118]. Also activation of microglia yielding increased release of proinflamatory interleukins may induce neurodegeneration through Wnt or p75 neurotrophin receptor-dependent signal transduction pathways [75, 119, 120]. On the other hand, anti-p75/Wnt antibodies or silencing respective genes, protected against excitoxicity/inflammation-induced loss of cholinergic neurons [54, 121].

Most of these cytotoxic signals directly affected activities of enzymes linked with energy and acetyl-CoA metabolism in humans as well as animal and cellular models of AD [13, 27, 96, 102, 104, 118].

Therefore, the key effects of diverse neurodegenerative signals were limitations in acetyl-CoA availability and its intraneuronal distribution, yielding preferential disintegration of cholinergic neurons in AD brains (Figs. 1, 2). In fact, suppression of PDHC and mitochondrial energy deficits preceded appearance of AD pathology in the brains of transgenic 3xTg-AD mice. However, microarray studies revealed that in late-dementive phase of AD the depression of oxidative phosphorylation was accompanied by up-regulation of those genes [96]. It suggests that down-regulation of energy metabolism in the course of AD might be a protective response of surviving neurons to decreased supply of glucose and oxygen taking place in this pathology [96].

#### Excitoxicity

Several common pathologies of ageing brain like general or focal hypoxia, hypoglyceamia, inflammation, or thiamine deficits, cause depolarization and Ca-overload of neuronal and non neuronal cells. Glutamatergic neurons constitute 50 % of all brain neurons and synaptic terminals [122]. Prolonged pathologic depolarization yields an excessive co-release of glutamate and Zn from brain “gluzinergic” terminals, triggering action potentials through NMDA, AMPA receptors and other voltage gated Ca channels located on postsynaptic neurons including cholinergic ones [123–125]. They cause dysfunction of postsynaptic neurons that, when aggravated, may evolve toward apoptosis or necrosis [126, 127].

Energy deficits also inhibit uptake of glutamate by adjacent astrocytes, due to the down-regulation of EAA, GLAST and GLT1 transporters and inhibition of their glutamine synthetase [128]. It increases glutamate and Zn levels within the synaptic clefts, yielding sustained depolarization of postsynaptic neurons, as well as adjacent astroglial and microglial cells [117]. The disruption of Ca^2+^ homeostasis affects enzymes linked with pathways involved in energy, neurotransmitter, and NO metabolism, which seem to be primary targets for excitotoxic signaling in the brain [37, 38, 118]. The Ca^2+^ excess in the mitochondrial compartment yields inhibition of PDHC due to activation of PDH kinase, being at least in part, the cause of acetyl-CoA deficits in subcellular compartments of cholinergic neuronal cells (Fig. 2) [27, 38, 129].

#### Zn Neurotoxicity

Zn co-released with glutamate from overexcited “gluzinergic” terminals may reach high, up to 0.3 and 1.0 mmol/L, concentrations in the synaptic cleft [113, 122]. Subsequently Zn is avidly taken up from the synaptic cleft by depolarized postsynaptic glial and neuronal cells including cholinergic ones, through the voltage gated, dilthiazem-sensitive Ca-channels and specific Zn-transporting proteins [38, 116, 130]. Extended depolarization brings about overloading of postsynaptic neurons with Zn, irrespective of expression of their cholinergic phenotype. Zn accumulated in postsynaptic cholinergic neurons was found to inhibit several enzymes of energy metabolism with pathophysiologically relevant potencies including: KDHC > NADP isocitrate dehydrogenase > aconitase > PDHC [37, 38]. The inhibitory interactions of Zn with lipoate binding sites of PDHC and KDHC could be reversed by the delayed application of lipoamide or chelating agents. On the contrary, inhibition of aconitase and isocitrate dehydrogenase appeared to be irreversible [37]. Such inhibitory pattern may impede therapeutic attempts aimed to rescue energy metabolism in excitotoxicity-affected brains. Moreover, in human AD brain cortex the alternation pattern of enzymes of energy metabolism appeared to be very similar to that found in Zn-treated SN56 cholinergic cells [13, 37]. It provides a circumstantial evidence on significance of aberrations in intraneronal distribution of Zn for the pathomechanism of AD.

The Zn-evoked inhibition of PDHC and other enzymes listed above, explains suppression of mitochondrial acetyl-CoA levels, and inhibition its metabolic flux through TCA cycle as a cause of ATP depletion and neuronal loss resulting from excitotoxic insults (Table 1; Fig. 2) [37, 38, 125, 126].

Subsequently, deficit of intramitochondrial acetyl-CoA would inhibit transfer of acetyl units to cytoplasm through different pathways (Fig. 1). Shortage of acetyl-CoA, in cytoplasm of cholinergic neurons, may decrease ACh level in this compartment, through ChAT equilibrium mechanism (Table 1; Fig. 2b, c, d) [66]. That in turn would reduce vesicular ACh pool available for quantal release. In this manner overload of postsynaptic cholinergic neurons with Zn could cause immediate depression of ACh synthesis, as well as its vesicular accumulation and release at early steps of neurodegeneration [46]. On the other hand, chronic excess of Zn could result in either death of some cholinergic neurons or depression ChAT activity in those surviving in these pathogenic conditions [37, 38]. Our studies revealed that differentiated cholinergic neuronal cells SN56, with high expression of the cholinergic phenotype, were more susceptible to Zn as well as other neurotoxic signals than those with low level of cholinergic metabolism (Table 1) [27, 38, 131]. This differential, phenotype-dependent susceptibility of septal cholinergic neurons to Zn-induced inputs could be directly linked with insufficient availability of acetyl-CoA in the mitochondrial compartment (Fig. 2a, b) [37, 38].

Zn itself may also increase Ca accumulation in the neurons, thereby facilitating opening PTP and release of cytochome c, caspases and other proapoptotic proteins from mitochondria [27, 116, 132]. This excess of Ca accumulated exclusively in the cytoplasmic compartment of cholinergic neuronal cells in Zn concentration-dependent pattern [38]. It could trigger PTP formation and aberrant compartmentalization of acetyl-CoA yielding cholinergic neurons injuries [37, 118, 124, 130].

#### NO Excess

Gutamate-Zn evoked increase of [Ca^2+^]/[calmodulin-Ca] in cytoplasmic compartments of postsynaptic neurons and adjacent glial cells markedly increases nNOS and eNOS activities, respectively [113, 117]. It seems however, that only increased expression of Ca-independent iNOS in the microglial/astroglial cells may contribute significantly to neurodegeneration. It has been demonstrated, that only iNOS-dependent activation may elevate the NO levels in the brain up to low micromolar, pathologically relevant, concentrations [113]. In fact, bacterial lipopolysaccharides could induce several-fold increase of NO synthesis by microglia [133]. On the other hand, fraction of NO produced by nNOS/eNOS may reach levels two orders of magnitude lower, and is likely to play a physiologic roles of “volume transmitter” and guanyl cyclase activator [117].

Peroxynitrite radicals were found to react with wide range of intracellular biomolecules linked with energy metabolism, glycolytic metabolism and several regulatory and transport or neurotransmitter functions, as well as with antioxidant systems. Excess of endogenous NO exerts rapid but reversible inhibition of cytochrome c oxidase and less potent one for other proteins of respiratory chain and ATP-synthetase, as well [113, 117]. However, NO may also inhibit earlier steps of energy metabolism including: PDHC, aconitase, isocitrate NADP-dehydrogenase, as well as KDHC [35, 77, 79]. Other enzymes of TCA cycle: succinate dehydrogenase, fumarase, and malate dehydrogenase were not affected by these conditions. That could cause deficits of acetyl-CoA and ATP in neuronal cells exposed to NO/ONOO^−^ excess [77, 79] Cholinergic neurons with residual expression of the cholinergic phenotype appeared to be more resistant to NO neurotoxicity than those with high expression of the cholinergic phenotype, apparently due to negligible demand for acetyl-CoA to support ACh synthesis in the former (Table 1; Fig. 2f) [35, 77, 79].

Lipoic acid or ALC were found to exert positive effects on viability of NO or Zn treated cholinergic SN56 cells through preservation of high acetyl-CoA levels in their subcellular compartments (Fig. 2) [27, 35]. However, delay in cytoprotectant application markedly diminished their efficacy, apparently due to instant, irreversible inactivation of aconitase by Zn and NO excess [27, 37, 38]. ChAT appeared to be resistant to direct, acute exposition to NO-excess. However, short exposition of differentiated SN56 cells to NO excess caused after 24 h delayed adaptative suppression of ChAT activity, apparently induced by shortages of acetyl-CoA in their mitochondrial and cytoplasmic compartments (Fig. 2b, c, d; Table 1) [15, 27, 77]. Additive effects of NO excess and Aβ were also observed (Table 1) [79]. Finally, alterations in activities of enzymes of energy metabolism in SNP-treated differentiated SN56 cells displayed pattern, similar to that reported for human AD brains [13, 35, 79]. Also cholinergic cells derived from basal forebrain were more sensitive to NO excess that those originated from brain stem, as indicated by their phospho ERK-2 and DNA fragmentation levels (Table 1) [134]. NO excess increased TUNEL reactivity, lactate dehydrogenase release and the decrease in ChAT activity in primary cholinergic neurons from medial septum, whereas those from brain stem were resistant to such conditions (Table 1) [135–137]. These data indicate that NO-derived radicals may exert differential cholinergic phenotype-specific neurotoxic effects in the brain.

#### Amyloid-β Neurotoxicity

Accumulation of fibrillar Aβ deposits in the interstitial compartments of the brain is a hallmark of AD and related amyloid encephalopathies [98, 99, 138]. The disruption of the cholinergic system and suppression of oxidative glucose metabolism were observed in brain regions with Aβ burden. Therefore, Aβ load was claimed to be the key factor triggering and supporting progress of the disease [98, 99]. However, degree of cognitive impairment correlated much better with loss of cholinergic neurons and impairment of oxidative metabolism than with Aβ burden in AD brains [13, 139]. In addition, some cholinergic encephalopathies develop without accumulation of Aβ but with impairment in brain energy metabolism [140–142]. Moreover, AD-type pathologic changes were found to affect about 80 % of individuals of age 65, but only small fraction of them suffers of dementia [143]. Therefore, Aβ overload in the brain should be treated as diagnostic and staging biomarker, and not as causative factor for this pathology [144, 145]. On the other hand, one cannot exclude possibility that Aβ, after reaching some hypothetical critical level, might aggravate preceding neurotoxic signals, triggering vicious cycle of neurodegeneration. For instance, extracellular fibrillar aggregates of Aβ were found to form autonomic or NMDA-linked high conductance Ca-channels in plasma membranes triggering Ca-dependent mechanisms of cytotoxicity, including depression of energy metabolism [115].

However, effective, cytotoxic concentrations of Aβ in most in vitro experimental conditions appeared to be an order of magnitude higher than those detected in AD brains varying from 0.5 to 4.0 μmol/kg [145–147].

Aβ_1–42_ in nontoxic, submicromolar concentrations inhibited PDHC and ACh synthesis/release in cultured primary septal neurons, without changing their ChAT activity [148]. This peptide also inhibited oxidative metabolism and decreased ATP contents in the PC12 cells [149]. Short time exposition to high 50 μmol/L Aβ inhibited glucose uptake and increased synthesis of free radicals in brain synaptosomes [150]. Differential, phenotype-dependent susceptibility of neurons to Aβ was reported by Olesen et al. [151]. They found, that immortalized raphe nucleus neurons, when differentiated to cholinergic phenotype with brain-derived neurotrophic factor became three times more sensitive to Aβ than those differentiated to serotoninergic phenotype with ciliary neurotrophic factor (Table 1) [151]. In addition, Aβ caused two times greater increase of free Ca in hippocampal than in cortical neurons [152]. Also injection of Aβ into rat nucleus basalis induced deficits of cholinergic neurons but increased activity of serotoninergic ones [153]. In cerebral neurons, Aβ inhibited cytochrome P450 epoxygenase, which metabolized arachidonic acid to neuroprotective epoxyeicosatrienoic acid, being inactive toward its cerebellar isoform. Therefore, cerebral and cerebellar neurons appeared to be prone and resistant to Aβ, respectively [154]. Differentiation of cultured embryonal rat brain neurons increased their susceptibility to Aβ [155]. It was due to activation of their cyclin-dependent kinase 5, which promotes tau phosphorylation, responsible for tubulin disintegration in Aβ-exposed neurons [155]. There is also known that energy deficits in different pathologies of aging brain induce β-processing of APP [94, 156]. On the contrary, dietary high energy substrates were found to reverse neuronal hyperactivity in transgenic AD mice [157]. Studies on differentiated cholinergic SN56 cells revealed that their greater susceptibility to Aβ could be caused by lower activity of PDHC, yielding decreased availability of acetyl-CoA in their mitochondria. In addition, high basal levels of Ca both in mitochondrial and cytoplasmic compartments of differentiated cells, could facilitate detrimental effects of Aβ (Fig. 2a, e; Table 1) [27, 55, 79]. Also increased expression of p75 receptors in differentiated cells could augment their sensitivity to Aβ in the presence of NGF [27, 54, 158]. There are also indications that Aβ may aggravate neurotoxic effects of other AD-linked toxins such as Al, Zn or peroxinitrine radicals (see “Zn Neurotoxicity”, “NO Excess”) [79, 113, 118, 159]. It has been found, that Aβ, Al or NO excess exerted cumulative suppressive effects on PDHC and aconitase activities, acetyl-CoA levels, cholinergic metabolism and viability cholinergic SN56 cells in culture (Figs. 1, 2) [27, 79, 118].

On the other hand, regional accumulation of Aβ in human AD brain did not correlate with loss of respective cognitive functions [139, 160]. Accordingly, in number of patients with heavy Aβ load, but with normal deposition of amyloid P in the brain cortex and hippocampus, no signs of dementia have been found. On the other hand, individuals with moderate Aβ accumulation and high content of amyloid P displayed typical symptoms of AD dementia [161].

Also in 3xTgAD mice cognitive impairment appeared before formation any Aβ and tau deposits in their brains [162]. Apparently, Aβ accumulation should be considered rather as an outcome than a trigger of AD neurodegeneration [145, 163]. However, it does not exclude possibility that accumulated Aβ might in combination with earlier cytotoxic signals aggravate end stages of cholinergic encephalopathies.

#### Transgenic Animal Models of Cholinergic Degeneration

Several transgenic mice (Tg) models have been developed to study mechanisms of Aβ-dependent neurodegeneration and investigate putative therapeutic approaches to AD. All of Tgs were designed by single or multiple inserts of mutated *APP*, *PS1* human gen(s) into mice germinal cells. If the above assumptions are correct, then Tg-AD mice should display: (1) Aβ accumulation in sensitive areas of the brain cortex in their advanced age, (2) functional and structural disruption of cholinergic neurons paralleling deficits of cognitive functions, (3) suppression of energy metabolism preceding alterations, quoted above.

1. There are significant discrepancies concerning accumulation of Aβ_1–42_ in brains of Tg animals. For instance, in brains of Tg2576 mice, Aβ_1–42_ levels reported by different authors varied from 0.13 through 0.94 μmol/kg to over 200 times higher concentrations of 30 μmol/kg of tissue (Table 1) [164–166]. What is interesting, the degree of cognitive impairment, and histochemical amyloid plaque densities were similar in all strains. Also, strikingly wide range of Aβ_1–42_ levels was reported for *post mortem* AD human brains [146, 167]. Such wide dispersion of patholologic values may, presumably result from still existing analytical problems concerning Aβ recovery, matrix effects, standardization, or specificity and avidity of used antibodies.
2. There are also significant differences between alterations in cholinergic marker levels in human AD and mice Tg brains. In human AD brains, significant decreases in ChAT activities and other cholinergic markers correlated with loss of cognitive functions (see “Alzheimer's disease”). On the other hand, no losses of ChAT activity and VAChT levels were observed in brains of demented Tg-AD or TS65DN mice despite evident cognitive deficits [168, 169]. However, decreases in ACh content and release rates were found in Tg-AD mentally impaired mice, despite of normal ChAT activity [166, 170].
3. The inhibition of glucose uptake and pyruvate utilization in FVB mice took place, which was likely to cause deficits of acetyl-CoA for energy and ACh production (Table 1) [171]. However, no suppressions in PDHC, aconitase, ICDH-NADP and KDHC activities were found in brains of 17 months Tg 2576 mice (Table 1) [165] (Szutowicz, Schliebs unpublished). They remain in contrast with findings in human AD brains, which revealed significant decreases in activities of all enzymes listed above [13]. Data presented here indicate that in Tg mice structural integrity of cholinergic neurons is better preserved than in human AD brains. Thus, suppression of ACh metabolism and dependent cognitive functions in Tg-mice is of functional character, resulting from relatively weak inhibition of glucose and pyruvate metabolism by accumulating Aβ, which is not deadly to cholinergic neurons [14, 171, 172].

### Thiamine Deficiency Encephalopathy

Animals and humans have no capacity of thiamine ring synthesis, which is the precursor of thiamine diphosphate (TDP). TDP is a cofactor of two key enzymes of energy metabolism PDHC and KDHC, providing acetyl-CoA for and limiting metabolic flux through TCA cycle, respectively. Therefore, thiamine deficits (TD) impair functions of all body tissues, yielding multi symptomatic and diverse clinical presentations of cholinergic transmission deficits. There are claims that, TD-induced inhibition of oxidative metabolism might facilitate and exacerbate amyolid plaque pathology [156, 173, 174]. In consequence, alcohol abuse, which is a most common worldwide cause of TD, should be considered as a risk factor for AD [175].

#### Energy and Acetyl-CoA Metabolism in Thiamine Deficient Brain

Early studies on pyrithiamine-TD rat brains reported either no change or slight decrease of acetyl-CoA levels [48, 176]. They however, addressed neither intercellular nor intracellular compartmentalization of this metabolite. So far, only few studies provided quantitative data on mitochondrial and cytoplasmic pools of acetyl-CoA in different cellular compartments in TD (Table 1, Figs. 1, 2) [36, 131].

In TD brains two major mechanisms seem to contribute to an early dysfunction and late loss of cholinergic neurons. They include: primary limitation of acetyl-CoA provision by unproductive TD-PDHC and secondary excitotoxic over-activation by glutamate-Zn released from energy-depleted glutamatergic neurons [37, 122]. The combination of TD-evoked, direct inhibition of PDHC and KDHC and Zn-induced excitotoxicity may aggravate acetyl-CoA and energy shortages in these conditions [36, 38, 128, 177]. In such conditions reductions in intramitochondrial levels of acetyl-CoA correlated with loses of cholinergic markers and viability of cholinergic neuronal cells (Fig. 2a, b, d) [15, 27, 36–38, 131].

There is well established, that the decrease of cytoplasmic acetyl-CoA in TD SN56 cells and brain nerve terminals resulted from limited availability of this metabolite in the mitochondrial compartment (Figs. 1, 2b, c). In consequence, lower rates of ACh release in TD neurons positively correlated with decreased concentration of acetyl-CoA in their cytoplasmic compartment (Fig. 2d) [38, 131]. These findings fit to a general rule that the rate of ACh synthesis/release depends on the availability of acetyl-CoA in cytoplasmic/synaptoplasmic compartment of cholinergic neurons, irrespective of neurotoxic signal (Figs. 1, 2c, d) [6, 27, 118]. However, unlike for AD or other neurotoxic conditions, acute TD altered ChAT activity neither in pyrithiamine-rat brain synaptosomes nor in amprolium-SN56 cells [38, 131, 176, 178]. These data indicate that, at least in early stages of TD, the structure of cholinergic neurons remained well preserved.

Also in this model of cholinergic pathology, neurons with high expression of the cholinergic phenotype appeared to be more susceptible to TD than those with residual cholinergic activity. There was in line with lower levels of mitochondrial acetyl-CoA in the DC (Fig. 2c; Table 1) [131]. Residual ACh metabolism in nondifferentiated cholinergic neurons was not affected by TD-induced shortages in mitochondrial and cytoplasmic acetyl-CoA (Table 1, Figs. 1, 2c, d, e) [130]. Other, in vivo observations on differential regional susceptibility of brain cholinergic neurons and energy metabolism to TD remain in accord with cell culture studies [1, 156, 179].

## Choline

Choline is another precursor of ACh, which is utilized in equivalent amounts with acetyl-CoA for the transmitter synthesis. Mammalian brain has limited capacity to synthesize its own choline. Therefore, this ACh precursor has to be transported from intravascular compartment across the blood brain barrier [180, 181]. Cholinergic neuron axonal terminals possess highest density CHT1 that are specific functional proteins for their axonal terminals [182, 183]. It has been suggested, that only choline transported by CHT1 is available for ACh synthesis.

In pathological conditions, prolonged depolarization of cholinergic terminals may inhibit CHT1-dependent transport of choline from extracellular space due to collapse of membrane potential. In such conditions, choline may originate from hydrolysis of structural membrane phospholipids by phospholipase D [181]. This choline pool could supplement ACh synthesis and maintain stable transmitter level during its excessive release in course of sustained depolarization of cholinergic neurons. However, if such conditions persist the phosphatidylcholine content in membranes of cholinergic neurons would decrease, resulting in their shrinkage and death [181]. That could contribute to particular susceptibility of cholinergic neurons to neurodegenerative insults [181]. On the contrary, the increase of extracellular choline to supraphysiological concentrations increased both basal and depolarization-evoked ACh release. It indicates, that choline taken up by low affinity system could also be used for transmitter synthesis and release. These observations rationalize use of choline supplementation as a method improving ACh metabolism in age–evoked disturbances of cholinergic system in the brain [184, 185]. Unlike acetyl-CoA, choline provision for ACh synthesis is not directly dependent on the energy production. However, correction of acetyl-CoA and energy deficits in aged rats by ALC supply in drinking water elevated choline uptake as well as ACh synthesis and release [85, 182].

On the other hand, choline-deficient diets induced deficits of this precursor both in cell membranes and in the ACh synthesizing compartment [186]. Lack of membrane integrity could cause the impairment of energy production in brain mitochondria, leading to increased vulnerability of cholinergic neurons to neurodegenerative signals along with appearance of behavioral deficits [186, 187]. Such reciprocal interactions between aberrant acetyl-CoA/energy and choline metabolisms may trigger vicious cycle of early deterioration of cholinergic neurons under various neurodegenerative conditions.

## Future Directions

Vast number of studies has been performed over the past three decades on the mechanisms of AD and other cholinergic encephalopaties. They employed diverse in vivo, in vitro models, and clinical studies focused mainly on disturbances in Aβ metabolism. Only small fraction of them focused on malfunctions of energy metabolism and their role in onset and progress of AD. The latter reveal decreases in PDHC and KDHC activities, which depress acetyl-CoA synthesis and utilization, in regions of brain cortex affected by this pathology [27, 118]. There is however a significant time gap between observations on acute, excitotoxic alterations in energy metabolism and late structural, irreversible changes in the neuronal network [188]. Therefore, several questions remain to be answered regarding time-course transition mechanisms between early, apparently reversible stages of inhibition of acetyl-CoA/energy production and delayed, irreversible phase of impairment of brain cholinergic system. To address this problem multiple time and neurotoxin dose-dependent studies on expression and postranlational modifications in enzymes/proteins of energy metabolism and intracellular signal transduction pathways should be executed. Further studies are also necessary on reciprocal, differential acute and chronic effects of AD pathology on energy/acetyl-CoA metabolism in dominating class of glutamatergic neurons as well as astro and microglial cells to link them with acute and prolonged abberations in their functions and integrity. They should provide a detailed map of regional and inter/intracellular compatrmentalization of acetyl-CoA in the brain. This might pave the path for studies on interventions aiming to preserve acetyl group metabolism, in manner adequate to early and late stages of neurodegeneration.
